# Plexiform neurofibromatosis of the bladder leading to cystectomy: A rare complication of von Recklinghausen disease

**DOI:** 10.1002/ccr3.7515

**Published:** 2023-06-13

**Authors:** Saina Paymannejad, Pardis Rafei Dehkordi, Reza Kazemi

**Affiliations:** ^1^ Department of Urology, School of Medicine Isfahan University of Medical Sciences Isfahan Iran

**Keywords:** bladder neurofibromatosis, cystectomy, Neurofibromatosis type 1, von Recklinghausen

## Abstract

We report a 20‐year‐old man with neurofibromatosis type 1 presenting with frequent episodes of suprapubic pain. The episodes started 6 months ago, occurred 1 h a day, and were not related to urination. A prostate‐sparing cystectomy with orthotopic diversion was performed. Histopathological assessment of the specimen confirmed bladder plexiform neurofibromatosis.

## Graphical abstract

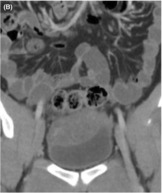

## 1 INTRODUCTION

Neurofibromatosis is a neurocutaneous genetic disorder caused by a mutation in the neurofibromin gene. The altered gene products play a role in the dysregulation of tumor suppression. Neurofibromatosis type 1 (NF1), neurofibromatosis type 2, and schwannomatosis are three types of the disease, which differ regarding the age of onset, clinical manifestations, and gene loci.^1^

NF1, also known as von Recklinghausen disease, is the peripheral type of the disease affecting several systems in the body. It manifests at birth or early childhood with classic symptoms of multiple hyperpigmented (café‐au‐lait) macules, intertriginous freckling, cutaneous neurofibromas, and varying levels of cognitive impairment. Although NF1 is mainly inherited as an autosomal dominant pattern, sporadic mutational change is also frequent.^2^ One of the rarest complications of NF1 is the involvement of the genitourinary tract, with the urinary bladder being the most frequently affected organ. Bladder neurofibroma should be inspected thoroughly as on the one hand, it has the potential for mimicking the characteristics of different malignant tumors, and on the other hand, it is threatened by the risk of malignant transformation.^3^

Regarding the very rare nature of bladder involvement in NF1 and the lack of sufficient data about its diagnostic and treatment approaches, formulating the diagnosis in the first place, and providing accordant management is of great importance. Therefore, this case report aims to lead the audience's mind toward bladder involvement as one of the rare but important complications of NF1, to be able to take timely relevant measures. Herein we report a case of NF1 with diffused bladder involvement in a 20‐year‐old man who presented with frequent episodes of suprapubic pain but did not complain of any urinary symptoms.

## 2 CASE PRESENTATION

A 20‐year‐old man was referred to the department of Urology, affiliated with Isfahan University of Medical Sciences, complaining of frequent episodes of suprapubic pain. The pain episodes had started 6 months ago, occurred once a day, lasted approximately 1 h, were accompanied by nausea and constipation, and were not related to urination. He denied any lower urinary tract symptoms (LUTS), gross hematuria, or significant weight loss since the beginning of the symptoms.

**FIGURE 1:**
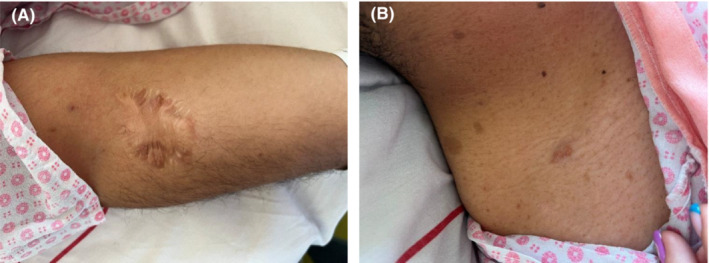
(A) Scar from the previous cutaneous neurofibromatosis tumor removal on the right forearm. (B) Numerous café‐au‐lait lesions on the neck and shoulder.

The patient had a past medical history of sporadic neurofibromatosis with no positive family history. He had previously undergone outpatient surgical removal of some of the cutaneous neurofibromatosis tumors on the forearm and back (Figure 1A), as well as an open surgical resection of a pelvic mass, which was ultimately diagnosed as neurofibroma upon the histopathological examination of the specimen.

A physical exam noted an overweight individual compatible with his age. All of the vital signs were within the normal range. There was mild tenderness in the suprapubic region and various café‐au‐lait lesions were observed on the abdomen, feet, shoulder, and forearms (Figure 1B). Other systems' examination was unremarkable. Laboratory data included a hemoglobin level of 14.5 g/dL, a total leukocyte count of 8600/μL with differential counts as neutrophils 71.8% and lymphocytes 25.4%, a serum creatinine level of 1 mg/dL and 1‐h erythrocyte sedimentation rate (ESR) of 8 mm/h. Analysis of the midstream urine sample revealed 1–2 WBC and 2–3 RBCs in each high‐power field and few bacteria. Besides, urinary nitrite, as well as urine culture after 24 h, were negative.

**FIGURE 2:**
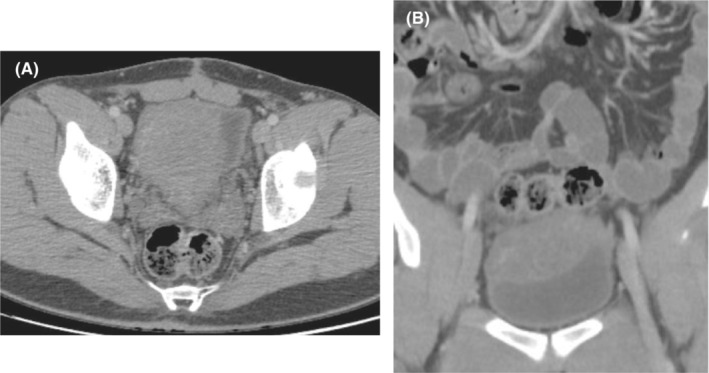
CT scan of the abdomen and pelvis. (A) Axial section showing a high attenuated lobular marginated mass occupying the bladder. Note the diffused thickening of the bladder wall. (B) Coronal section confirming aforementioned changes.

A multi‐detector computed tomography (MDCT) of the abdomen and pelvis with contrast medium injection was performed to further investigate the cause of the chronic suprapubic pain. The results revealed diffused bladder wall thickening, as well as a high attenuated lobular marginated mass in size of 83*76 mm involving the superior and posterior parts of the bladder protruding into the lumen and causing a pressure effect (Figure 2).

Since the patient had previously experienced visceral neurofibroma, bladder neurofibroma was considered our first differential diagnosis; however, other bladder tumors could not be ruled out without histopathological confirmation.

A pre‐operative biopsy for detecting the tumor type was not indicated since tumor excision was necessary due to the significant impact of chronic pain on the patient's quality of life. Given the tumor's large size, an open laparotomy approach was conducted in order to excise the tumor and investigate other adjacent structures. Under general anesthesia, in the supine position, the bladder was approached by a mid‐line sub‐periumbilical laparotomy. After resecting the adjacent fascia, a large mass with invasion to the dome, trigone, and posterior part of the bladder was detected. In addition, the bladder appeared deformed and tumorous with several adhesions to the rectum and peri‐vesical fat; therefore, resection of the primary tumor alone did not seem to be effective, and we intended to perform a prostate‐sparing cystectomy along with orthotopic urinary diversion, using approximately 60 cm of the ileum with proper vascular base.

**FIGURE 3:**
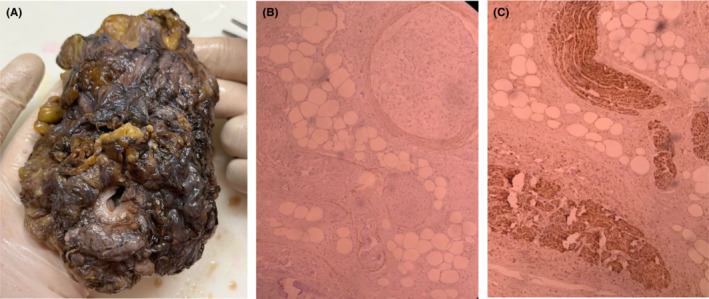
(A) Gross specimen of the resected bladder. Note the deformed shape and multiple neurofibromas within the bladder wall, (B, C) Histopathological examination of the surgical specimen with hematoxylin and eosin staining demonstrating a diffused and plexiform growth pattern in the bladder wall, composed of peripheral nerves, axons, Schwann's cells, collagen bundles, and fibroblasts.

The resected specimen was sent to the pathology department. Macroscopic evaluation demonstrated a urinary bladder measuring 14*8*4.5 cm on the external surface containing serosal and muscular walls and multiple masses with the largest diameter of 2 cm (Figure 3A). Histopathological analysis revealed a diffused and plexiform growth pattern composed of peripheral nerves including axons, Schwann's cells, collagen bundles, and fibroblasts (Figure 3B,C). Immunohistochemical staining was positive for S100 and negative for epithelial membrane antigen (EMA). The findings were highly in favor of plexiform neurofibroma with bladder wall involvement. Besides, no evidence of malignancy was detected in the pathological examination.

The postoperative course was uneventful and the patient was discharged 5 days later in good general condition. On an outpatient follow‐up visit after 6 months, the pain was completely resolved and he did not complain of any urinary symptoms.

## 3 DISCUSSION

Bladder involvement in NF1 is very rare and mostly affects the age group of 7 to 28 years^4^ with a ratio of 3:1 male predominance.^5^ Neurofibromas often arise from the nervous ganglia of the bladder wall, especially the vesico‐prostatic plexus.^6^ They could either involve a discrete region of an individual nerve and present as a solitary tumor or, as occurred in our patient, implicate several nerve fascicles, extend beyond the nerve branches and form a plexiform neurofibroma, leading to diffused infiltrative process of the bladder wall.^1^

Clinical manifestations are often associated with the mass effect and include irritative LUTS, hematuria, and recurrent urinary tract infection.^7^ Since the distribution of the vesical nervous plexus is closely associated with the trigone, ureters, and urethra, formation of neurofibromas along these structures could result in obstructive symptoms and urinary retention as well. This is mostly observed in plexiform neurofibromas involving multiple adjacent nerve branches and are the presenting picture of approximately two‐third of bladder neurofibromatosis.^8^ Nonspecific symptoms due to compression of surrounding tissue could also be present.^9^ On the other hand, Baugh et al. described an asymptomatic 19‐year‐old male with an isolated 2.4 cm bladder neurofibroma, which was incidentally discovered on CT‐scan.^10^ In a cohort study conducted by Tonsgard et al, 65% of the patients with abdomino‐pelvic plexiform neurofibroma were asymptomatic.^11^ As a result, the type and severity of the symptoms vary significantly among individuals depending on the location, size, and number of tumors. One of the tricky aspects of our case was the absence of any urinary symptoms despite the occupation of trigon, diffused plexiform infiltration, and larger size of the tumor.

No definitive criteria have been established for diagnosing bladder neurofibromatosis; however, the diagnosis is initially formed based on clinical and radiological findings and later confirmed by histopathology. Both magnetic resonance imaging (MRI) and CT‐scan could be used to identify the tumor. Data regarding the representative radiological findings is scares. Castillo et al. described bladder wall thickening as the most distinctive radiological feature.^12^ Shonnard et al. reported that both simple and plexiform bladder neurofibromas present in CT‐scan as homogenous masses with attenuation values of 20–30 HU, which is similar to the adjacent skeletal muscle, and slightly enhances with contrast medium injection.^8^, ^13^ The low density could be due to the high lipid content, high water content in the mucosal matrix and zones of hypo‐cellularity within the mass.^14^ In our case, despite the diffused plexiform involvement of the bladder wall, we could not detect the smaller neurofibromas on CT‐scan; however, radiological characteristics of the larger mass were consistent with previously described findings.

MRI is believed to be the imaging of choice, especially in cases of abdomino‐pelvic involvement. It is superior to CT‐scan regarding its ability in distinguishing benign and malignant lesion as well as identifying small neurofibromas with rather atypical features.^13^ Due to the detection of the large neurofibroma on CT scan and the necessity of its removal, we did not need to perform MRI as it could not change our management approach.

Histopathologically, bladder involvement usually exhibits diffuse proliferation of uniform neurofibromas containing multiple nests of medium‐sized spindle cells with elongated wavy nuclei and myxoid stroma. In addition, mast cells could be present in small numbers within the stroma.^15^

In general, there is a 30% chance that a neurofibroma would undergo malignant degeneration^16^; however, bladder neurofibromatosis is almost always a benign process and malignancy has been reported in 5%–10% of the literature.^17^ One of the life threatening tumors associated with NF1 is malignant peripheral nerve sheath tumor (MPNST), which is often precursed by the plexiform types or solitary tumors of a sizeable nerve existing for an extended period of time.^18^ Despite the low rate of malignant transformation, bladder neurofibromas can mimic the characteristics of some other malignant tumors. A differential diagnosis of leiomyosarcoma, ganglioneuroma and paraganglioma should always be well thought‐out in cases of solitary lesions.^19^ As a result, malignancy either as a differential diagnosis or a transformative process of the existing neurofibroma should always be considered. Clinically, malignant tumors are associated with unexplained persistent pain, rapid growth and texture alteration from soft to hard.^1^ Moreover, asymmetry of the lesions, infiltrative margins, heterogeneity, and significant enhancement following contrast injection are radiological findings in favor of malignancy.^14^ In our patients, considering malignancy was obliged due to the chronic persistent suprapubic pain, radiological enhancement and lobulated margins of the tumor; however, no pathological evidence in favor of malignancy was discovered.

Regarding the variable clinical manifestations and the difference in severity and type of bladder involvement, every patient must be approached individually in terms of management modalities. Surgical excision of the tumor seems to be the most adopted method.^20^ In cases of progressive growth of the tumor, involvement of a large part of the bladder, or the presence of incapacitating symptoms, cystectomy is inevitable. Our patient was managed with a prostate‐sparing cystectomy. We chose an orthotopic urinary diversion due to the patient's younger age and the better prognosis of this technique for preserving sexual function.^21^

In conclusion, contrary to the majority of the literature, the distinguishing feature of our case was the absence of any urinary symptoms despite the great occupation of the bladder. One should always rule out malignancy in cases of bladder involvement. This article emphasizes the importance of timely identification of bladder neurofibromatosis and having a holistic vision while choosing the appropriate management option.

## DECLARATION OF FIGURES' AUTHENTICITY

The presented figures have been created by the authors who confirm that they are original and not copied from elsewhere.

## AUTHOR CONTRIBUTIONS

**Saina Paymannejad:** Investigation; validation; visualization; writing – original draft; writing – review and editing. **Pardis Rafei Dehkordi:** Investigation; methodology; project administration; visualization; writing – original draft. **Reza Kazemi:** Conceptualization; methodology; project administration; resources; supervision.

## ACKNOWLEDGMENTS

The authors would like to express their gratitude to the study patient who signed written consent for publishing this information.

## FUNDING INFORMATION

This report did not receive any specific grant from funding agencies in the public, commercial, or not‐for‐profit sectors.

## CONSENT

Written informed consent was obtained from the patient to publish this report in accordance with the journal's patient consent policy.

## Data Availability

Data will be available on request.
